# Prevalence of General and Central Obesity and Associated Factors among North Korean Refugees in South Korea by Duration after Defection from North Korea: A Cross-Sectional Study

**DOI:** 10.3390/ijerph15040811

**Published:** 2018-04-20

**Authors:** Yoon Jung Kim, Sin Gon Kim, Yo Han Lee

**Affiliations:** 1 Division of Endocrinology and Metabolism, Department of Internal Medicine, Hallym University Hangang Sacred Heart Hospital, Beodeunaru-ro 7-gil, Yeongdeungpo-gu, Seoul 07247, Korea; yjkim99@hallym.or.kr; 2 Division of Endocrinology and Metabolism, Department of Internal Medicine, Korea University College of Medicine, Anam-dong 5-ga, Seongbuk-gu, Seoul 02841, Korea; k50367@korea.ac.kr; 3 Department of Preventive Medicine, Konyang University College of Medicine, 158, Gwanjeodong-ro, Seo-gu, Daejeon 35365, Korea

**Keywords:** North Korean refugees, obesity, central obesity, associated factors

## Abstract

Previous studies on obesity status among North Korean refugees (NKRs) have been limited. We investigated mean body mass index (BMI), waist circumference (WC), and general and central obesity prevalence among NKRs in South Korea (SK) by duration after defection from North Korea (NK), using cross-sectional data of the North Korean Refugee Health in South Korea (NORNS) study and compared these data with a sample from the general South Korean population (the fifth Korea National Health and Nutrition Examination Survey). The prevalence of general and central obesity among NKRs with duration after defection from NK of less than five years were lower than among South Koreans, except for central obesity among NKR females (obesity prevalence, 19% (12–27%) vs. 39% (34–44%) for NK vs. SK males (*p* < 0.001) and 19% (14–24%) vs. 27% (24–29%) for NK vs. SK females (*p* = 0.076); central obesity prevalence, 13% (6–19%) vs. 24% (20–29%) for NK vs. SK males (*p* = 0.011) and 22% (17–28%) vs. 20% (18–22%) for NK vs. SK females (*p* = 0.382)). The prevalence of general and central obesity among NKRs with duration after defection from NK (≥10 years) were comparable to those of South Koreans in both genders (obesity prevalence, 34% (18–50%) vs. 39% (34–44%) for NK vs. SK males (*p* = 0.690) and 23% (18–29%) vs. 27% (24–29%) for NK vs. SK females (0.794); central obesity prevalence, 21% (7–34%) vs. 24% (20–29%) for NK vs. SK males (*p* = 0.642); 22% (17–28%) vs. 20% (18–22%) for NK vs. SK females (*p* = 0.382)). Male sex, age and longer duration after defection from NK (≥10 years) were positively associated with obesity. As for central obesity, age was the only independently associated factor. NKR females with duration after defection from NK of less than five years had comparable central obesity prevalence to South Korean females in spite of a lower BMI, which suggests that we need further monitoring for their metabolic health among NKRs in SK.

## 1. Introduction

Since the Korean War of the 1950s, North Korea (NK) and South Korea (SK) have evolved different socioeconomic systems. NK embraced communism and became impoverished in the 1990s. In contrast, SK introduced a market economy system and achieved rapid economic growth for decades. The economic gap between NK and SK has continually increased because of a series of natural disasters and the collapse of communism in NK [1]. North Korean refugees (NKRs), experiencing chronic food shortage in NK, fled to China or other transit countries and some finally resettled in SK. The total number of NKRs in SK increased from 1000 in 2001 to 30,000 in 2016 [2]. Thus, the successful resettlement of NKRs is an important issue in SK. With a shared ethnic, cultural and linguistic background, North Koreans in their home country are characterized by lower height and body weight than South Koreans due to chronic food shortage [3,4,5]. However, after defection from NK and resettlement in SK, they may experience catch-up weight gain with exposure to environment rich in food [6]. As the time since defection from NK increases, NKRs may become exposed to the Westernized diet and sedentary lifestyle of SK. Thus, not only infectious diseases but also chronic diseases, such as obesity and cardiovascular disease, have become important issues. Korea was a nation with shared ethnic background for thousands of years and NK and SK separated only for six decades since the Korean war. Therefore, comparison between the two Koreans offers a good opportunity to study the effect of environment on human health. However, few studies on chronic diseases such as obesity and cardiovascular disease among NKRs have been performed [6,7,8].

Previous studies on immigrants who have migrated from low- or middle-income countries to high-income countries have shown that the body mass index (BMI) of immigrants who have resided longer in host country was higher than that of recently arrived immigrants [9,10]. As acculturation proceeds, immigrants become integrated into the social environment of the host country. Indeed, the BMI of immigrants significantly increases 10–15 years after migration, by which time their prevalence of obesity is close to or greater than that of host population [11]. BMI is the most frequently used measure of obesity in studies on immigrants, but it cannot distinguish between lean and fat mass. Waist circumference (WC) is good indicator of abdominal visceral fat distribution and a strong predictor of diabetes mellitus and cardiovascular disease [12]. As Asians have higher proportions of body fat and central obesity than American or European population with similar BMI, it is meaningful to investigate the change of WC as well as BMI among NKRs in SK by duration after defection from NK [13].

In this study, we investigated changes in mean BMI, WC and prevalence of general and central obesity among NKRs in SK by duration after defection from NK, using cross-sectional data of the North Korean Refugee Health in South Korea (NORNS) study. We also compared these data with a sample from the general South Korean population. We further investigated factors associated with general and central obesity among NKRs.

## 2. Materials and Methods

We used the data from the NORNS study to investigate the changes in mean BMI and WC among NKRs by duration after defection from NK [14]. The NORNS study has been conducted at Korea University Anam hospital in SK since October 2008 to comprehensively investigate the health status of NKRs and is still ongoing. Participants were recruited by the Hana Center, which advertised the study on the Internet and contacted each NKR by telephone. Eligible individuals (NKRs ≥ 20 years of age) voluntarily participated in the study. From October 2008 to March 2017, a total of 932 NKRs (males = 192, females = 740) participated and detailed protocols have been described previously [14]. The study was approved by the Institutional Review Board of Korea University Anam hospital (approval number: Ed08023).

Height, body weight and WC were measured by trained technicians and graduate students using standard methods [14]. Height and body weight were measured using an automatic height and weight measuring instrument. WC was measured in triplicate at the midpoint between the lower ribs and iliac crest. BMI was defined as body weight in kilograms divided by square of height in meters. We defined being underweight, normal weight, overweight, and obesity as BMI of <18.5, 18.5–22.9, 23.0–24.9 and ≥25 kg/m^2^, respectively, according to the Asian cutoff points [15]. Central obesity was defined as WC of ≥90 cm for males and ≥85 cm for females according to the Korean cutoff points [16]. We excluded participants for whom data were missing or unreliable (BMI ≤ 12 or ≥50 kg/m^2^; WC ≤ 50 or ≥150 cm). The final sample consisted of 917 participants (Figure S1).

We also collected data on demographic characteristics including age, sex, education level, current income in SK and number of family members in SK. To evaluate the migration index among NKRs, data on the time of defection from NK, time of arrival in transit countries and time of arrival in SK were collected. We further categorized age as 20–39, 40–59 and ≥60 years and duration after defection from NK as <5, 5–10 and ≥10 years. We defined higher education as more than college graduation. Health-related lifestyle factors such as current smoking, alcohol consumption and exercise were measured. Frequent alcohol consumption was defined as more than one bottle of alcohol per week. Regular exercise was defined as vigorous activity more than 1 hour per week. In the NORNS study, a doctor who had defected from NK conducted one-on-one interviews with all NKRs who participated in the study to enhance the completeness of the participants’ questionnaires.

We investigated the changes in mean BMI, WC and prevalence of general and central obesity among NKRs by duration after defection from NK and compared these data with a sample from the fifth Korea National Health and Nutrition Examination Survey (KNHANES) conducted in 2012. A total of 1834 South Korean subjects, who were age- and sex-matched to the NKR participants at a ratio of 1:2, were randomly sampled from the fifth KNHANES. The KNHANES is a representative sample of SK assessing health status and health-related behaviors at a national level using a multi-stage probability sampling method [17].

Descriptive analyses of sociodemographic and anthropometric characteristics were performed by gender. All expression levels are given as either numbers (%, proportions) or mean ± standard deviations. We also performed stepwise binary logistic regression analyses using general and central obesity as dependent variables to identify factors associated with general and central obesity among NKRs. Adjusted odds ratios (AORs) and corresponding 95% confidence interval (CIs) were calculated. Statistical significance was assessed at the α = 0.05 level. All analyses were performed using SAS 9.2 software (SAS Institute, Cary, NC, USA).

## 3. Results

The general characteristics of the NKRs are shown in Table 1. Of the 917 participants, 728 (79%) are females and the mean age of total participants is 43.8 ± 12.6 years. Most of the NKRs are between 20 and 60 years of age. The proportion of NKRs who are underweight is 3.9% for males and 3.1% for females. The proportion of NKRs who are overweight or obese accounted for 49% for males and 41% for females. The obesity prevalence is 22.6% for males and 19.7% for females, without any gender difference. The prevalence of central obesity in males is lower than that in females (13.3% vs. 21.4%, males vs. females, *p* = 0.014). The mean duration after defection from NK is 5.5 years for males and 7.6 years for females, and the mean duration of stay in transit countries is 2.0 years for males and 4.2 years for females. Not only NKR females stay longer in transit countries but also have longer duration after defection from NK compared to males. As for health-related lifestyle factors, a greater proportion of males than females are current smokers, drink alcohol frequently and exercise regularly.

### 3.1. The Mean BMI and Obesity Prevalence among NKRs by Duration after Defection from North Korea

The mean BMI and obesity prevalence among NKR males with duration after defection from NK of less than five years are lower than those of South Korean counterparts (Table 2, BMI, 22.6 kg/m^2^ (22.1–23.1 kg/m^2^) vs. 25.2 kg/m^2^ (24.2–26.1 kg/m^2^), NK vs. SK; obesity prevalence, 19% (12–27%) vs. 39% (34–44%), NK vs. SK). The mean BMI and obesity prevalence among NKR females with duration after defection from NK of less than five years are lower than those of South Korean counterparts with marginal statistical significance (Table 2, BMI, 22.7 kg/m^2^ (22.3–23.1 kg/m^2^) vs. 23.4 kg/m^2^ (23.0–23.9 kg/m^2^), NK vs. SK; obesity prevalence, 19% (14–24%) vs. 27% (24–29%), NK vs. SK). In both genders, the mean BMI and obesity prevalence among NKRs with duration after defection from NK (≥10 years) are comparable to those of South Koreans.

The trend of the change in mean BMI by duration after defection from NK vary by gender. The mean BMI among NKR males increase with duration after defection from NK (Table 2, <5 years, 5–10 years, ≥10 years, 22.6 kg/m^2^ (22.1–23.1 kg/m^2^), 23.0 kg/m^2^ (22.1–23.8 kg/m^2^), 24.1 kg/m^2^ (23.1–25.0 kg/m^2^), respectively, p for trend = 0.012). In contrast to males, the mean BMI among NKR females do not differ by duration after defection from NK (Table 2, <5 years, 5–10 years, >10 years, 22.7 kg/m^2^ (22.3–23.1 kg/m^2^), 22.6 kg/m^2^ (22.3–23.0 kg/m^2^), 23.0 kg/m^2^ (22.6–23.4 kg/m^2^), respectively, *p* for trend = 0.386).

### 3.2. The Mean WC and Central Obesity Prevalence among NKRs by Duration after Defection from North Korea

NKR males with duration after defection from NK of less than five years have a lower WC and prevalence of central obesity than South Korean counterparts (Table 3, WC, 80.1 cm (78.6–81.6 cm) vs. 84.8 cm (83.7–85.9 cm), NK vs. SK; central obesity prevalence, 13% (6–19%) vs. 24% (20–29%), NK vs. SK). By contrast, the mean WC and central obesity prevalence among NKR males with duration after defection from NK (≥10 years) are comparable to those of South Korean counterparts (Table 3, WC, 84.0 cm (81.2–86.7 cm) vs. 84.8 cm (83.7–85.9 cm), NK vs. SK; central obesity prevalence, 21% (7–34%) vs. 24% (20–29%), NK vs. SK). In contrast to NKR males, NKR females with duration after defection from NK of less than five years have the mean WC and central obesity prevalence comparable to South Korea counterparts (Table 3, WC, 78.1 cm (77.0–79.3 cm) vs. 77.3 cm (76.7–78.0 cm), NK vs. SK; central obesity prevalence, 22% (17–28%) vs. 20% (18–22%), NK vs. SK).

The trend of change in mean WC by duration after defection from NK vary depending on gender. The mean WC among NKR males increase by duration after defection from NK (Table 3, <5 years, 5–10 years, >10 years, 80.1 cm (78.6–81.6 cm), 81.0 cm (78.4–83.7 cm), 84.0 cm (81.2–86.7 cm), respectively, *p* for trend = 0.032). Among NKR females, the mean WC show no difference by duration after defection from NK (Table 3, <5 years, 5–10 years, ≥10 years, 78.1 cm (77.0–79.3 cm), 77.9 cm (76.7–78.1 cm), 78.5 cm (77.4–79.7 cm), *p* for trend = 0.717).

### 3.3. Factors Associated with Obesity among NKRs

Table 4 shows the results of stepwise binary regression analyses to identify factors affecting obesity among NKRs. After adjusting for multiple sociodemographic variables (Model 4), male sex (male vs. female; AOR, 1.87; 95% CI, 1.07–3.26), older age (>60 years vs. 20–39 years; AOR, 6.16; 95% CI, 3.39–11.17) and longer duration after defection from NK (≥10 years vs. <5 years; AOR, 1.68; 95% CI, 1.08–2.62) are positively associated with obesity. Smoking is negatively associated with obesity (current smoking vs. no smoking; AOR, 0.42; 95% CI, 0.19–0.92).

### 3.4. Factors Associated with Central Obesity among NKRs

Table 5 shows the results of stepwise binary regression analyses to identify factors affecting central obesity among NKRs. After adjusting for multiple sociodemographic variables (Model 4), older age (≥60 years vs. 20–39 years; AOR, 14.90; 95% CI, 7.92–28.02) is positively associated with central obesity. Neither duration after defection from NK nor gender is associated with the risk of central obesity.

## 4. Discussion

We investigated the trend of changes in mean BMI, WC and prevalence of general and central obesity among NKRs in SK by duration after defection from NK and compared these data with those of South Korean counterparts. The mean BMI, WC and prevalence of general and central obesity among NKRs with duration after defection from NK of less than five years were lower than those of South Korean counterparts, except for central obesity among NKR females. As for central obesity, NKR females with duration after defection from NK of less than five years have comparable mean WC and central obesity prevalence to South Korea counterparts. In both genders, NKRs with duration after defection from NK (≥10 years) had comparable BMI, WC and prevalence of general and central obesity to those of South Korean counterparts.

In this study, prevalence of underweight among NKRs in SK was 3.9% for males and 3.1% for females, which was lower value than those in previous study among NKRs [18]. The difference in proportion of underweight between two studies may be due to the fact that mean duration after defection from NK in previous study was shorter than in this study (2.8 years vs. 7.0 years, respectively) [18]. In our study, the prevalence of obesity among NKRs in SK is about five times higher than those of North Koreans in their home country, suggesting that NKRs experienced a significant increase in body weight after defection from NK (22.6% vs. 4.1%, in SK vs. in NK for males, 19.7% vs. 4.7%, in SK vs. in NK for females, respectively) [4,6,7,19].

It could be an acculturation process that mean BMI, WC and prevalence of general and central obesity among NKRs with duration after defection from NK (≥10 years) were comparable to those among South Koreans. This is consistent with previous studies that immigrants’ body weight increase significantly during the 10 to 15 years after their arrival in host country and obesity prevalence among immigrants is close to that of host population after 10 to 15 years of immigration [11,12]. In our study, duration of residence in the host country is shorter than previous studies because we used duration after defection from NK as a parameter for investigating changes of BMI and WC.

Among NKR males, mean BMI and WC increased by duration after defection from NK. In contrast to NKR males, NKR females did not show the trend of increasing mean BMI and WC by duration after defection from NK. The reason that the change in mean BMI and WC by duration after defection from NK differs by gender is because the prevalence of obesity in males is higher than those of females in SK. In SK, males have higher prevalence of obesity than female [20,21]. Males and females have different attitudes toward weight perception and different practices for weight control because social pressure against obesity is stronger for females than for males in SK [22]. The fact that the mean BMI and WC in NKR females did not increase by duration after defection from NK could be a process of assimilation into South Korean society.

As for factors associated with obesity, NKRs with duration after defection from NK (≥10 years) had 1.7 times higher risk for obesity than NKRs with duration after defection from NK of less than five years, consistent with previous studies that there is a significant positive relationship between BMI and duration of residence in the host country [10,11].

The finding that NKR males were more likely to be obese than females is consistent with previous studies on Korean American immigrants [23]. Gender difference in the effects of residence duration on obesity have been shown to vary by ethnicity and host country. For example, Hispanic female immigrants in the United States have a greater tendency to be obese than male immigrants [24,25]. As previously mentioned, the tendency toward a higher risk of obesity among NKR males in SK may be a part of acculturation process into South Korean society.

Age is a well-known risk factor for obesity [20,23]. NKRs over 60 years of age were six times more likely to be obese than those who were 20–39 years of age.

As for health-related lifestyle factors, smoking was the only factor negatively associated with obesity. Inconsistent with previous studies, regular exercise did not have any effect on the obesity status in our study [26,27]. Further research is needed to determine how physical activity affects the obesity status of NKRs, particularly among NKRs who defected from NK (≥10 years).

As for central obesity, NKR females with duration after defection from NK of less than five years had similar mean WC and central obesity prevalence with South Korean females. The only factor independently associated with central obesity was age, which is a well-known risk factor for central obesity, especially for females [28,29]. The risk for central obesity among NKR females over 60 years of age was about 15 times higher than that among females aged 20–39 years of age. We found no significant association between duration after defection from NK and risk for central obesity, suggesting that a certain degree of central obesity was present even prior to defection, especially in older NKR females. NKR females may have more children than South Korean females, which may explain the higher prevalence of central obesity [30]. According to subgroup analysis of NKR females, both age and multiparity (gave birth to more than three children) were independently associated with central obesity (Table S1). NKR females with duration after defection from NK of less than five years had similar WC as South Korean females despite their lower BMIs, which suggested that BMI may not be representative of the metabolic health among NKR females.

We investigated change in mean BMI and WC among NKRs in terms of post-defection period from NK, including not only duration of stay in SK but also in transit countries, because mean BMI and body weight among NKRs continued to increase during resettlement in SK through transit countries (Table S2).

There were several limitations to our study. First, we did not apply scientific sampling methods to the study population. Random sampling of NKRs in SK remains difficult owing to the small number of NKRs in SK, and NKRs tended to conceal their identities. However, our participants did not differ from those who participated in a national survey conducted by the South Korean government in terms of sex ratio, age distribution, mean duration of stay in transit countries and place of birth in NK [6]. Second, the cross-sectional nature of the study prevented us from inferring any causal relationship. Third, in contrast to NKR females, the sample size of NKR males was too small to enable reliable comparison analyses with South Korean counterparts. Fourth, the relatively short duration of stay in SK among NKRs (3.4 ± 3.2 years, mean ± standard deviation) was too brief to investigate the association between duration of residence and change in BMI and WC. Fifth, NKR had different duration of stay in transit countries, which may have affected their BMI and WC. Lastly, we considered duration after defection from NK as acculturation scale, without including language proficiency or affinity with South Korean society. We did not include language proficiency or affinity to South Korean society because NKRs use the same language and share the same cultural background with South Koreans.

Despite these limitations, this is the first study to investigate the prevalence of general and central obesity among NKRs in SK by duration after defection from NK and compare these data with a sample of the general South Korean population. Future research is needed to investigate the effects of changes in dietary habits and physical activity after defection from NK on BMI among NKRs. A prospective study is also needed to examine longitudinal changes in BMI and WC and their influence on the occurrence of cardiovascular disease and diabetes mellitus among NKRs in SK.

## 5. Conclusions

The prevalence of general and central obesity among NKRs with duration after defection from NK of less than five years were lower than those South Korean counterparts, except for central obesity among NKR females. In both genders, NKRs with duration after defection from NK (≥10 years) had comparable prevalence of general and central obesity as South Koreans, which suggests the acculturation process of NKRs to South Korean society. As for central obesity, NKR females with duration after defection from NK of less than five years have comparable central obesity prevalence as South Korean females, despite having lower BMI, which suggests the need for further monitoring on metabolic health among NKRs in SK.

## Figures and Tables

**Table 1:** General characteristics of North Korean refugees (NKRs).

Variables	Male	Female	*p*-Value
Number	189 (20.6)	728 (79.4)	
Age (years)	46.1 ± 12.9	43.3 ± 12.5	0.049
20–39	63 (33.3)	327 (44.9)	
40–59	92 (48.7)	306 (42.0)	
≥60	34 (18.0)	94 (12.9)	
Height (cm)	166.0 ± 6.6	154.1 ± 5.0	
Body weight (kg)	63.3 ± 8.8	53.9 ± 7.4	
Waist circumference (cm)	81.1 ± 7.8	78.2 ± 8.8	
Prevalence of central obesity *	24 (13.3)	151 (21.4)	0.014
Body mass index (kg/m^2^)	23.0 ± 2.6	22.7 ± 2.9	0.299
Weight status ^†^			
Underweight	6 (3.3)	26 (3.6)	
Normal weight	86 (47.5)	395 (54.9)	
Overweight	48 (26.5)	155 (21.5)	
Obesity	41 (22.6)	142 (19.7)	
Duration after defection from NK (years)	5.5 ± 4.7	7.6 ± 4.8	<0.001
<5	113 (61.1)	235 (33.8)	
5–10	32 (17.3)	221 (31.8)	
≥10	41 (21.7)	142 (19.3)	
Duration of stay in transit countries (years)	2.0 ± 2.8	4.2 ± 3.9	<0.001
Health-related lifestyle factors			
Current smoker	91 (48.0)	9 (1.1)	<0.001
Frequent alcohol consumption ^‡^	134 (70.8)	444 (61.0)	<0.001
Regular exercise ^§^	110 (58.2)	355 (48.8)	0.019
Socioeconomic status			
Higher education	50 (26.4)	133 (18.2)	0.28
Monthly income > 10^6^ (KRW)	54 (44.2)	194 (39.9)	0.056
Living alone	62 (39.2)	266 (41.4)	0.076

Categorical variables are given as number (%), Continuous variables are given as mean ± standard deviation. * Prevalence of central obesity: definition, male, ≥90, female, ≥85 (cm). ^†^ Weight status: underweight, <18.5; normal weight, 18.5–22.9; overweight, 23.0–24.9; obese, ≥25 (kg/m^2^). ^‡^ Frequent alcohol consumption: more than one bottle of alcohol per week. ^§^ Regular exercise: vigorous activity more than one hour per week. Higher education: more than college graduate. Abbreviations: NK, North Korea; KRW, Korean won.

**Table 2:** The mean BMI and obesity prevalence among NKRs compared with South Koreans.

Variables	Male	Female
Mean BMI (kg/m^2^)		
Defection period (years)		
<5	22.6 (22.1–23.1)	22.7 (22.3–23.1)
5–10	23.0 (22.1–23.8)	22.6 (22.3–23.0)
≥10	24.1 (23.1–25.0)	23.0 (22.6–23.4)
*p* for trend	0.012	0.386
South Koreans ^†^	25.2 (24.2–26.1)	23.4 (23.0–23.9)
Prevalence of obesity (%) *		
Defection period (years)		
<5	19 (12–27)	19 (14–24)
5–10	23 (7–38)	17 (12–22)
≥10	34 (18–50)	23 (18–29)
*p* for trend	0.180	0.202
South Koreans ^†^	39 (34–44)	27 (24–29)

* Prevalence of obesity: definition, BMI ≥ 25 kg/m^2^. ^†^ South Koreans: sample from fifth Korea National Health and Nutrition Examination Survey conducted in 2012. Abbreviations: BMI, body mass index; NKRs, North Korean refugees.

**Table 3:** The mean WC and central obesity prevalence among NKRs compared with South Koreans.

Variables	Male	Female
Mean WC (cm)		
Defection period (years)		
<5	80.1 (78.6–81.6)	78.1 (77.0–79.3)
5–10	81.0 (78.4–83.7)	77.9 (76.7–78.1)
≥10	84.0 (81.2–86.7)	78.5 (77.4–79.7)
*p* for trend	0.032	0.717
South Koreans ^†^	84.8 (83.7–85.9)	77.3 (76.7–78.0)
Prevalence of central obesity (%) *		
Defection period (years)		
<5	13 (6–19)	22 (17–28)
5–10	6 (0–15)	20 (15–25)
≥10	21 (7–34)	22 (17–28)
*p* for trend	0.206	0.801
South Koreans ^†^	24 (20–29)	20 (18–22)

* Prevalence of central obesity: definition WC of ≥90 cm for males and ≥85 cm for females. ^†^ South Koreans: sample from fifth Korea National Health and Nutrition Examination Survey conducted in 2012. Abbreviations: WC, waist circumference; NKRs, North Korean refugees.

**Table 4:** Result of logistic regression analyses on obesity.

Variables	Model 1 (*n* = 898)	Model 2 (*n* = 863)	Model 3 (*n* = 863)	Model 4 (*n* = 765)
Male vs. Female	1.01 (0.67–1.52)	1.11 (0.72–1.69)	1.43 (0.86–2.39)	1.87 (1.07–3.26)
Age (years)				
40–59 vs. 20–39	4.52 (2.93–6.98)	4.92 (3.14–7.70)	4.77 (3.04–7.49)	5.23 (3.21–8.53)
≥60 vs. 20–39	6.27 (3.71–10.60)	6.47 (3.77–11.12)	6.01 (3.48–10.4)	6.16 (3.39–11.17)
Defection period (years)				
5–10 vs. <5		1.05 (0.67–1.64)	1.05 (0.67–1.65)	1.13 (0.70–1.83)
≥10 vs. <5		1.56 (1.04–2.35)	1.52 (1.00–2.29)	1.68 (1.08–2.62)
Current smoker			0.50 (0.24–1.05)	0.42 (0.19–0.92)
Frequent alcohol consumption *			1.09 (0.76–1.56)	1.15 (0.78–1.68)
Regular exercise ^†^			1.28 (0.90–1.82)	1.32 (0.90–1.92)
Low income ^‡^				1.22 (0.77–1.94)
Low education ^§^				1.38 (0.86–2.21)
Living alone				1.04 (0.70–1.55)

Values are expressed as adjusted odds ratio (95% confidence interval). Model 1: adjusted for sex, age. Model 2: Model 1 + adjusted for duration after defection from North Korea. Model 3: Model 2 + adjusted for health-related lifestyle factors (current smoker, alcohol consumption, exercise). Model 4: Model 3 + adjusted for socioeconomic status (income, education, number of family member). * Frequent alcohol consumption: more than one bottle of alcohol per week. ^†^ Regular exercise: vigorous activity more than one hour per week. ^‡^ Low income: monthly income lower than 100,000 Korean won. ^§^ Low education: less than college graduate.

**Table 5:** Result of logistic regression analyses on central obesity.

Variables	Model 1 (*n* = 884)	Model 2 (*n* = 849)	Model 3 (*n* = 849)	Model 4 (*n* = 753)
Male vs. Female	2.50 (1.52–4.11)	2.45 (1.47–4.08)	1.92 (1.06–3.51)	1.51 (0.79–2.89)
Age (years)				
40–59 vs. 20–39	5.42 (3.31–8.87)	5.58 (3.37–9.24)	5.55 (3.34–9.22)	5.37 (3.16–9.12)
≥60 vs. 20–39	17.79 (10.06–31.45)	18.08 (10.10–32.36)	17.58 (9.76–31.67)	14.90 (7.92–28.02)
Defection period (years)				
5–10 vs. <5		0.93 (0.58–1.47)	0.93 (0.59–1.49)	1.04 (0.63–1.70)
≥10 vs. <5		1.15 (0.74–1.77)	1.12 (0.72–1.73)	1.19 (0.74–1.92)
Current smoker			0.52 (0.21–1.29)	0.40 (0.15–1.05)
Frequent alcohol consumption *			1.07 (0.73–1.56)	1.10 (0.74–1.64)
Regular exercise ^†^			1.05 (0.72–1.53)	1.06 (0.72–1.57)
Low income ^‡^				1.05 (0.60–1.84)
Low education ^§^				1.35 (0.82–2.21)
Living alone				1.03 (0.68–1.56)

Values are expressed as adjusted odds ratio (95% confidence interval). Model 1: adjusted for sex, age. Model 2: Model 1 + adjusted for duration after defection from North Korea. Model 3: Model 2 + adjusted for health-related lifestyle factors (current smoker, alcohol consumption, exercise). Model 4: Model 3 + adjusted for socioeconomic status (income, education, number of family members) * Frequent alcohol consumption: more than one bottle of alcohol per week. ^†^ Regular exercise: vigorous activity more than one hour per week. ^‡^ Low income: monthly income lower than 100,000 Korean won. ^§^ Low education: less than college graduate.

## Supplementary Materials

The following are available online at http://www.mdpi.com/1660-4601/15/4/811/s1, Figure S1: Flow diagram of the study, Table S1: Result of logistic regression analysis on central obesity among NKR females, Table S2: Mean body weight and BMI in North Korea, transit countries and the day on survey among NKRs.
