# Supplementary material for: Prevalence of General and Central Obesity and Associated Factors among North Korean Refugees in South Korea by Duration after Defection from North Korea: A Cross-Sectional Study
Source: Int J Environ Res Public Health. 2018 Apr 20;15(4):811. doi: 10.3390/ijerph15040811 (PMC5923853; doi:10.3390/ijerph15040811)
Supplement: Supplementary file 1 [file ijerph-15-00811-s001.pdf]

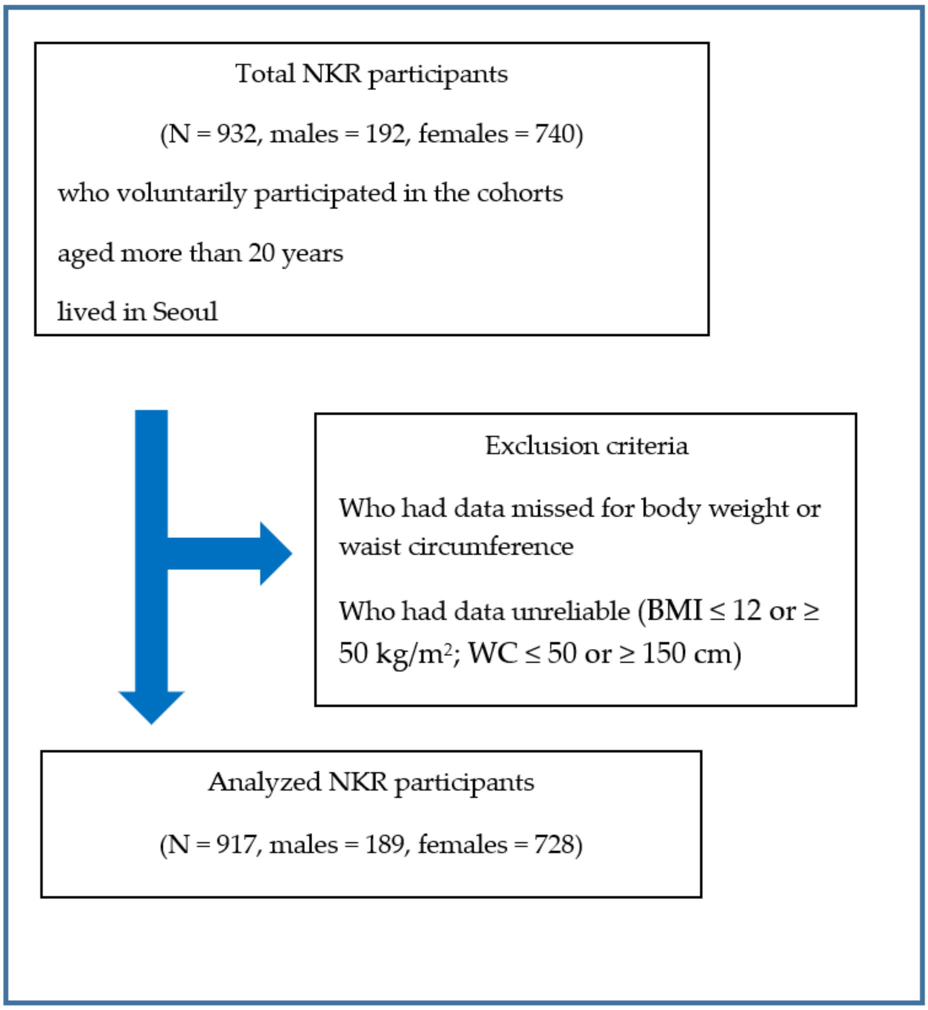

Figure S1. Flow diagram of the study. NKR: North Korean refugee.

Table S1. Result of logistic regression analysis on central obesity among NKR females.

Variables	Model 1 (n = 514)	Model 2 (n = 493)	Model 3 (n = 493)	Model 4 (n = 447)
Age (years)				
40–59 vs. 20–39	4.68 (2.47–8.88)	4.58 (2.41–8.73)	4.47 (2.34–8.54)	4.13 (2.12–8.04)
>60 vs. 20–39	12.45 (5.5–28.18)	12.44 (5.43–28.50)	12.51 (5.41–28.90)	10.24 (4.10–25.57)
Multiparity (≥ 3 children)	2.09 (1.22–3.59)	2.02 (1.16–3.52)	2.02 (1.15–3.52)	1.94 (1.06–3.56)
Defection period (years)				
5–10 vs. <5		1.06 (0.59–1.89)	1.00 (0.55–1.80)	0.99 (0.53–1.85)
≥10 vs. <5		1.12 (0.64–1.95)	1.10 (0.63–1.92)	0.97 (0.53–1.78)
Current smoking			2.31 (0.27-19.65)	2.22 (0.27–18.49)
Frequent alcohol drinking *			1.23 (0.76–2.00)	1.31 (0.79–2.20)
Regular exercise †			1.16 (0.72–1.85)	1.19 (0.72–1.96)

Low income ‡	1.88 (0.98–3.61)
Low education §	1.17 (0.61–2.25)
Living alone	0.90 (0.51–1.56)

Values are expressed as adjusted odds ratio (95% confidence interval). Model 1: adjusted for sex, age.Model 2: Model 1 + adjusted for duration after defection from North Korea.Model 3: Model 2 + adjusted for health-related lifestyle factors (current smoking, alcohol drinking, exercise). Model 4: Model 3 + adjusted for socioeconomic status (income, education, number of family member) * Frequent Alcohol drinking: more than one bottle of alcohol per week. † Regular exercise: vigorous activity more than one hour per week. ‡ Lowe income: monthly income lower than 100,000 Korean won. § Lower education: less than college graduate.

Table S2. Mean body weight and BMI in North Korea, transit countries and the day on survey among NKRs.

Variables	In NK	Arrival in SK	On Survey in SK
Body weight (kg)			
Male	60.2 (9.3)	62.1 (8.7)	63.2 (8.8)
Female	51.2 (7.1)	53.0 (9.2)	53.9 (7.4)
BMI (kg/m^2)			
Male	21.7 (3.0)	22.4 (3.9)	23.0 (2.6)
Female	21.6 (3.0)	22.3 (3.9)	22.7 (2.9)

Abbreviations: BMI: body mass index; SK: South Korea; NK: North Korea.
